# Hysterectomy and Adnexal Procedures by Vaginal Natural Orifice Transluminal Endoscopic Surgery (VNH): Initial Findings From a Korean Surgeon

**DOI:** 10.3389/fmed.2020.583147

**Published:** 2021-02-22

**Authors:** Myeong-Seon Kim, Joseph J. Noh, Tae-Joong Kim

**Affiliations:** ^1^Department of Obstetrics and Gynecology, College of Medicine, St. Vincent's Hospital, The Catholic University of Korea, Seoul, South Korea; ^2^Department of Obstetrics and Gynecology, Samsung Medical Center, Sungkyunkwan University School of Medicine, Seoul, South Korea

**Keywords:** hysterectomy, natural orifice transluminal endoscopic surgery, NOTES, vaginal NOTES, VNH

## Abstract

**Objective:** To evaluate feasibility and safety of hysterectomy and adnexal procedures by vaginal natural orifice transluminal endoscopic surgery (vNOTES).

**Study Design:** This is a prospective observational study at a tertiary center and teaching University hospital. We enrolled prospectively 34 patients with benign diseases sequentially.

**Results:** We measured baseline characteristics, surgical data, and pain score (VAS) after surgery. We surveyed before/after surgery. The time of port installation and each stage of surgery was measured. The learning curve was assessed through the graph according to the number of operations using linear and logarithmic regression curve estimation. The complications of surgery were investigated. The median age of the patients was 47.5 years (38–73). Median BMI was 22.4 (18.2–30.0). 20 cases of leiomyoma, four cases of adenomyosis, three cases of uterine prolapse, four cases of endometrial hyperplasia, and three cases of CIN were diagnosed. The median uterine weight was 180.0 g. The median port-installation time was 15.0 min (range, 4–35 min) and median total operation time was 85.5 min (range 43.0–132.0). Complications occurred in three patients. Two cases of bladder injury happened during vesicovaginal space dissection before the installation of the Wound Retractor (WR). One patient underwent transumbilical single-port surgery because of late-onset postoperative bleeding on the 13th postoperative day. The mean postoperative VAS scores were 3.36 immediately after surgery and 3.06, 2.79, and 2.45 at 6, 12, and 24 h after surgery, respectively. In continuous variable analysis, we detected a correlation between port-installation time and postoperative VAS ≥4 (pain score as need for medication). Based on a learning curve, port-installation time and total operation time appeared to reach the proficiency level by the 10th case.

**Conclusions:** Although there were three complications, vNOTES offers advantages to patients and surgeons. More surgical techniques will be developed in vNOTES.

## Precis

Vaginal natural orifice transluminal endoscopic surgery is a feasible and safe surgical technique on patients with gynecologic benign disease.

## Introduction

Laparoscopic hysterectomy (LH) and vaginal hysterectomy (VH) are minimally-invasive hysterectomy procedures with less pain, less visible scarring, less likelihood of postoperative adhesion formation, lower risk of developing postoperative infections, and faster recovery compared with hysterectomy through laparotomy (1). One of the advantages of the laparoscopic approach in comparison with the vaginal approach is that surgeons are able to explore the whole abdominal cavity before and after hysterectomy procedures. This provides opportunities for surgeons to remove pelvic adhesions by adhesiolysis prior to hysterectomy if needed as well as a chance to look for potential bleeding sites after the vaginal cuff is closed. In vaginal hysterectomy, however, the surgeons cannot examine the intraabdominal cavity once the vaginal cuff closure is done. Another advantage of the laparoscopic approach is that it enables surgeons to perform adnexal surgery whereas the vaginal approach limits surgical manipulations in the adnexae.

Since the early 2000s, natural orifice transluminal endoscopic surgery (NOTES) in gynecologic surgery has been performed (2, 3). NOTES is an operation in which surgeons approach into the abdominal cavity by creating an opening through the vagina. In 2012, vaginal NOTES (vNOTES) was reported as a feasible surgical technique for hysterectomy (3). vNOTES does not create an abdominal wound, which greatly enhances cosmetic outcomes and also avoids potential wound complications such as infection and herniation (4). Notably, vNOTES allows for unlimited ovarian and adnexal access, which is limited in VH. vNOTES thus enables visual exploration in the abdominal cavity.

For the first time in South Korea, we introduced vNOTES hysterectomy (VNH) in our hospital, which typically performs laparoendoscopic single-site surgery (LESS) or transumbilical single-port surgery, because we believed that vNOTES could give more benefits to patients and surgeons. The present study sought to report the initial data on our surgeries regarding patient response to this new surgical technique as reflected by a perioperative questionnaire. We also discuss the benefits and limitations of vNOTES from our initial experience.

## Materials and Methods

### Patients

After gaining approval from the Institutional Review Board (IRB) (SMC 2018-06-011-001), we prospectively enrolled 34 patients from April 2018 to June 2019 at Samsung Medical Center. Eligible patients included those with benign diseases such as leiomyoma, adenomyosis, uterine prolapse, and precancerous diseases such as cervical intraepithelial neoplasia (CIN) and endometrial hyperplasia. The surgeon (T-J Kim) performed bimanual pelvic examination and selected patients who demonstrated movable uterus. Patients with a narrow vaginal canal or any evidence of severe pelvic adhesion around the uterus were excluded. All surgical procedures were performed by an experienced gynecologic surgeon (T-J Kim).

### Surgical Techniques

**Figure 1:**
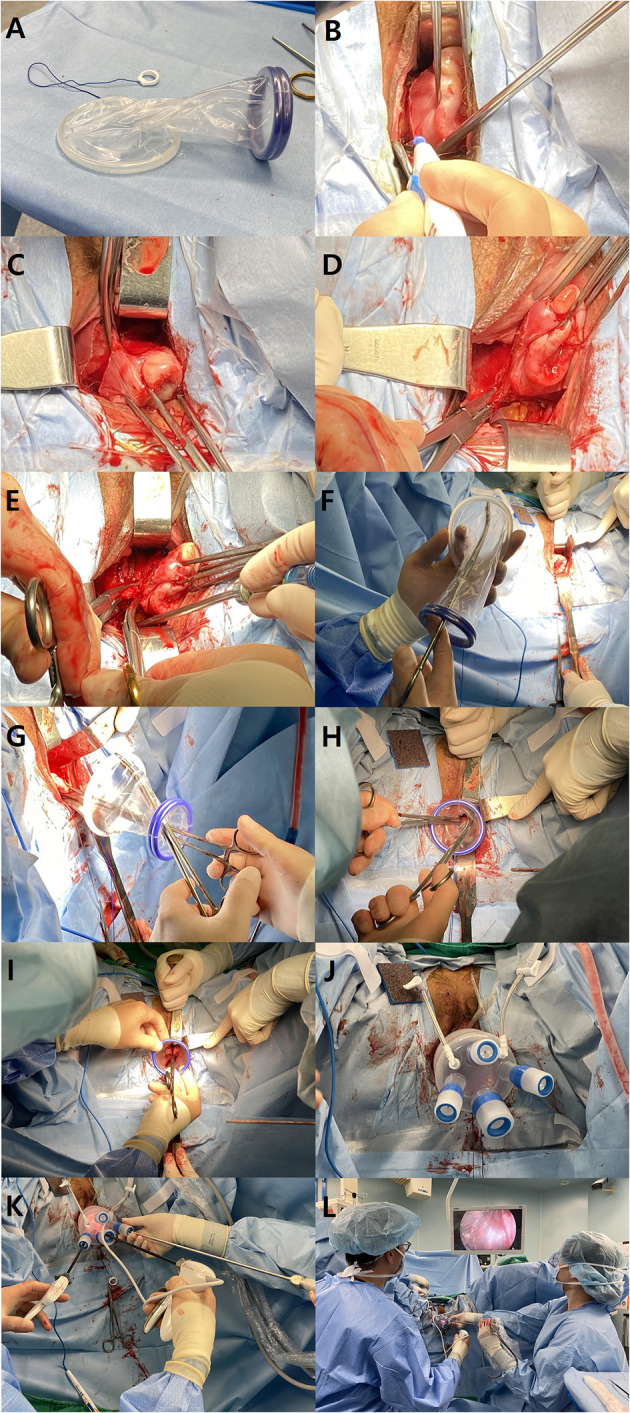
Procedures of vNOTES. **(A)** The wound retractor consists of two elastic rings with different diameters (6 and 9 cm). The larger ring is inserted and hooked at the vesicovaginal space and posterior cul-de-sac. **(B)** The anterior and posterior lips of the cervix are grasped together with two tenaculum forceps. A circumferential incision is made at the junction of the vagina and cervix. **(C)** The right bladder pillar is coagulated and cut with a monopolar Bovie electrocauterization. **(D)** The right uterosacral ligament is ligated with a suture ligature. **(E)** Following the right lateral wall of the uterus cephalad, the uterine artery is ligated with a suture ligature along with the cardinal ligament. **(F)** The larger ring of the wound retractor is grasped with a curved Kelly to facilitate the insertion into the peritoneum. The Kelly forceps are passed through the wound retractor, and the ring of the wound retractor is grasped inwards. **(G)** The cervix is grasped by tenaculum forceps that are also passed through the wound retractor. **(H)** The inner ring (larger diameter) is inserted into the peritoneum by gentle manipulation of the curved Kelly forceps. **(I)** The outer right (smaller diameter) is rolled up in order to tighten the wound retractor. **(J)** Multi-channel platform is installed on the wound retractor. **(K)** Three channels are used: one for a 30° 5 mm laparoscopy, one for laparoscopic grasper and one for an advanced energy device. **(L)** The position of the operator (left) and assistant (right) holding the camera.

All patients underwent the same standard preparation prior to surgery. Prophylactic antibiotic was administrated 30 min before incision. After general anesthesia and endotracheal intubation, the patient was placed in the Trendelenburg position with lithotomy. A 12-Fr Foley catheter was inserted. The operation began with anterior and posterior colpotomy as it is performed in conventional VH. The anterior and posterior lips of the cervix were grasped together with two tenacula. A circumferential incision was made at the junction of the vagina and cervix. The vesicovaginal space was created using blunt and sharp dissection. The bladder was displaced upward from the region of dissection by inserting a right-angle retractor into the vesicovaginal space. The rectum was displaced downward from the dissected region with a right-angle retractor after the posterior cul-de-sac was exposed by a culdotomy. Both uterosacral ligaments were ligated as well as both uterine arteries. The Wound Retractor (WR) and single-port platform (LapSingle, Sejong Medical Co., Ltd., Republic of Korea) were installed (Figure 1).

CO_2_ gas was used for pneumoperitoneum. CO_2_ pressure was set between 10 and 11 mmHg, which is lower than for conventional laparoscopic surgery. The total amount of CO_2_ used during the operation was measured.

We used a 30°-angled 5 mm endoscopy (Karl Storz GmbH & Co. KG, Tuttlingen, Germany) and laparoscopic instruments such as suction and irrigator, short grasper (ENDOPATH® Grasper 5 mm, Ethicon US, LLC), and Enseal® G2 Tissue Sealer (45 cm length and curved tip, Ethicon US, LLC).

We established the single-port platform at the vagina irrespective of the opening of the bladder peritoneum. The grasper held the cervix and pushed the cervix upward to make the operating space in the lateral side of the uterus. The Enseal® cut the remaining structures upward from the isthmus level. The round ligaments were the last remaining structure for hysterectomy. The resected uterus was taken out through the vagina after the single-port platform was detached from the WR. We then set up the single-port platform on the WR and continued endoscopic surgery for risk-reducing salpingectomy or salpingo-oophorectomy. We checked surgical sites to ensure hemostasis and irrigated the whole pelvis to remove any remaining blood clot. After removing the single-port platform and WR, we closed the vaginal cuff with our conventional method by suturing (5).

### Questionnaire

Postoperative cosmesis and surgical satisfaction were assessed using a questionnaire. The questionnaire was modified from the questionnaire used in a previous study, which had linguistic validation (6). After developing the questionnaire, additional linguistic validation was performed by two bilingual authors of this study.

The questionnaire used seven questions to evaluate surgical site pain and gas pain, the advantages of vNOTES, satisfaction with the surgery, changes in sexual life satisfaction after surgery, willingness to recommend the surgery to others (after surgery), and willingness to pay for vNOTES (after surgery). The patients answered the questionnaire four times: before surgery, 1 week after surgery, 6 weeks after surgery, and 3–6 months after surgery.

The questionnaire began to be used from the 15th patient. At the very initial phase of the implementation of this new surgical procedures, establishing the safety of the protocols was the most primary concern for the surgical team. Therefore, we focused on developing safe procedures rather than surveying patient satisfaction. The questionnaire was adopted from the 15th patient and the remaining patients all completed it during each outpatient visit.

### Treatment Protocol

Prophylactic antibiotic was administered preoperatively using a single dose of parenteral cefazolin and postoperatively using a single dose of cefazolin. After surgery, patients received NSAIDs as a pain management when needed. Visual analog scale (VAS) of pain was measured immediately after operation and then 6, 12, and 24 h after operation. Patients were discharged on the postoperative day (POD) 2 according to hospital policy if there were no complications. Patients visited the outpatient department at 1 week, 6 weeks, and 3–6 months after discharge. Twenty patients responded to the questionnaire on each outpatient visit.

### Data Analysis

Continuous variables in normal distribution such as age, BMI, and uterine weight are given as mean (SEM), and non-normal distribution data and discrete variables such as parity are given as median and range. The correlation between the data was analyzed using Spearman's correlation and independent samples *T*-test. The learning curve was obtained using regression curve estimation. Statistical analysis was performed using SPSS 24.0 for Windows (IBM Corp, Armonk, NY, USA).

## Results

Thirty four patients underwent vNOTES between April 2018 and June 2019. The baseline characteristics of the patients are shown in Table 1. Twenty six patients (76.5%) had a history of at least one vaginal delivery. Six patients had a history of at least one Cesarean delivery: three had Pfannenstiel's incision scar and three had lower midline incision scar. Three patients had received appendectomy and one had a history of laparoscopic cholecystectomy.

**Table 1:** Patient characteristics (*N* = 34).

**Variable**	***n***	**Range or %**
Median age (years)	47.5	38–73
Median BMI (kg/m^2^)	22.5	18.2–30.0
Previous vaginal delivery	26	76.5
Previous surgical procedure	10	29.4
**Type of previous surgical procedure**		
Cesarean section (Pfannenstiel's incision)	3	8.8
Cesarean section (low midline incision)	3	8.8
Appendectomy	3	8.8
Laparoscopic cholecystectomy	1	2.9

*BMI, Body mass index*.

The surgical outcomes are shown in Table 2. Thirteen cases of leiomyoma, four cases of adenomyosis, three cases of uterine prolapse, four cases of endometrial hyperplasia, and three cases of CIN (Grade 3) were diagnosed. The median uterine weight was 180.0 g. The median port-installation time was 15.0 min (range, 4–35 min) and median total operation time was 85.5 min (range 43.0–132.0). There was one case of failure to port-installation and there were three cases of conversion; two were vaginal total hysterectomy (VTH) and one was laparoendoscopic single-site surgery (LESS).

**Table 2:** Surgical outcomes (*N* = 34).

**Variable**	***n***	**Range or %**
Median EBL (mL)	100.0	20.0–500
Median Hb changes (g/dL)	1.85	0.2–4.5
Median hospital stay (days)	3	2–8
**Diagnosis**		
Leiomyoma	20	58.8
Adenomyosis	4	11.8
Endometrial hyperplasia	4	11.8
Cervical intraepithelial neoplasia (Grade 3)	3	8.8
Uterine prolapse	3	8.8
Median uterine weight (g)	180.0	50.0–670.0
Median port-installation time (min)^a^	15.0	4–35
Median total-operation time (min)	85.5	43.0–132.0
**Conversion of surgical method**		
VTH	2	5.8
LESS	1	2.9
**Complications**		
Bladder injury	2	7.70
Late, hemoperitoneum	1	3.85

a *There was one failure case of port-installation, in that case, we measured time until tried*.

Complications occurred in three patients. Two cases of bladder injury happened during vesicovaginal space dissection before the installation of the WR. One patient underwent transumbilical single-port surgery because of late-onset postoperative bleeding on the 13th postoperative day.

The mean postoperative VAS scores were 3.36 immediately after surgery and 3.06, 2.79, and 2.45 at 6, 12, and 24 h after surgery, respectively (Table 3A). In continuous variable analysis related to VAS measured immediately after the operation (Table 3B), there was no correlation between VAS and age, body weight, BMI, uterus weight, or total operation time. We detected a correlation between port-installation time and postoperative VAS ≥4 (pain score as need for medication).

Table 3**(A)** Postoperative pain analysis. **(B)** Variables related to VAS measured immediately after operation.

**Table:** 

**Time**	**Mean**	**Median VAS (range)**
**(A)**		
Immediately after surgery	3.36	3.0 (2–7)
6 h after surgery	3.06	3.0 (2–5)
12 h after surgery	2.79	3.0 (1–5)
24 h after surgery	2.45	2.0 (0–5)

**Table:** 

**Variable**	**4+** **VAS**	**N**	**Median**	**Range**	***P*****-value**
**(B)**					
Age	No	25	47.0	38–73	0.814
	Yes	9	48.0	44–64	
Body weight (kg)	No	25	55.4	46.30–76.10	0.901
	Yes	9	59.4	52.90–66.50	
BMI	No	25	22.3	18.18–28.60	0.314
	Yes	9	22.7	19.45–29.99	
Uterus weight (g)	No	25	210.0	50.0–670.0	0.230
	Yes	9	155.0	52.0–443.0	
Port-installation time (min)	No	25	15.0	4.0–27.0	0.013
	Yes	9	25.0	12.0–35.0	
Total operation time (min)	No	25	85.0	43.0–132.0	0.610
	Yes	9	94.0	57.0–125.0	

**Figure 2:**
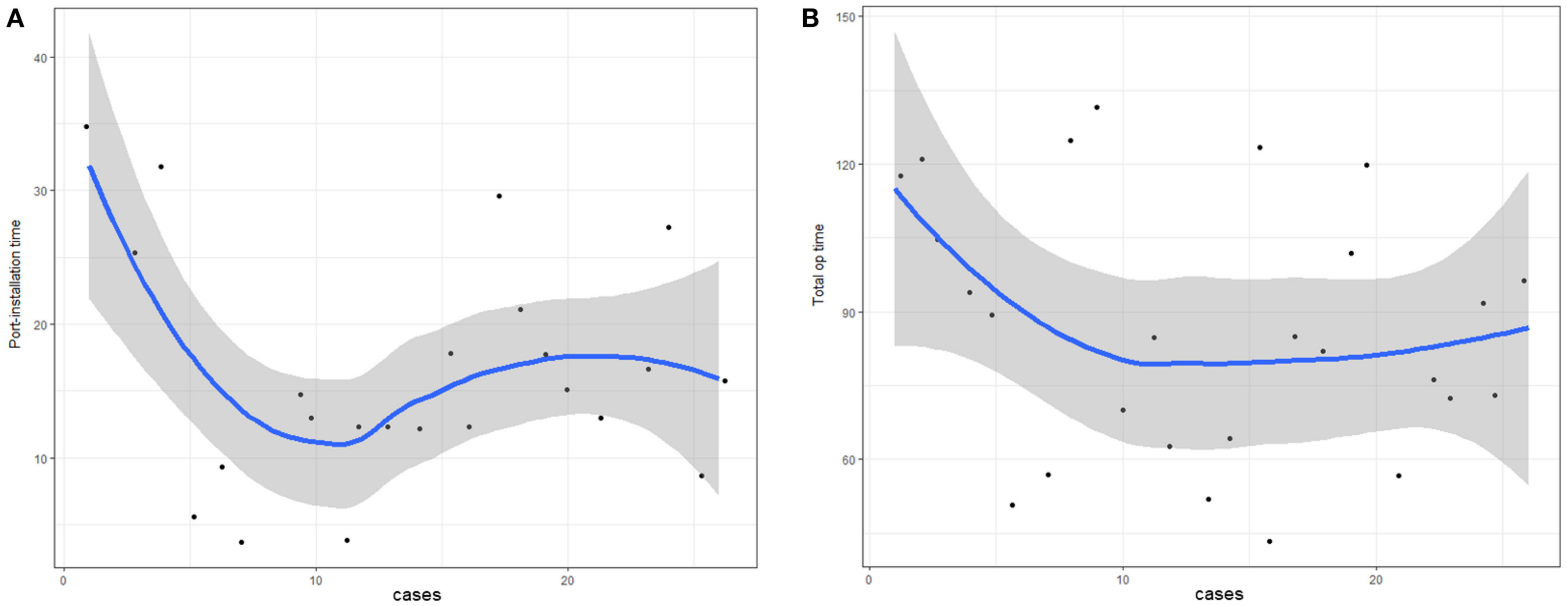
Learning curve of **(A)** port-installation time and **(B)** total operation time.

A learning curve based on the operation time of initial cases was created (Figure 2). Both learning curves (of port-installation time and total operation time) dropped sharply after the first five cases. Port-installation time and total operation time appeared to close to the proficiency level by the 10th case.

The survey was conducted in the last 20 patients (Table 4). In VAS of gas pain, low abdominal pain, and vaginal pain, 40, 30, and 45% of patients answered as no pain, respectively. In the survey at 1 week after surgery, overall surgical pain score was low and tolerable. In particular, none of the patients responded more than four points for overall vaginal pain. Nineteen patients responded as 10 points and one patient responded to nine points on the willingness to re-try the surgery, to recommend the surgery to friends, and overall satisfaction with vNOTES. With the exception of six patients who were not sexually active, 14 patients replied that their sexual satisfaction was altered 3–6 months after surgery. On the survey conducted before the surgery, seven of the 14 patients (50%) expected that sexual satisfaction would not change after surgery and the other seven patients (50%) expected it would decrease. On the survey 3–6 months after the surgery, two patients responded that sexual satisfaction improved and three patients responded no change. Six patients responded decreased sexual satisfaction. Sexual satisfaction is difficult to evaluate objectively because there are many factors affecting it.

**Table 4:** Summary of the survey after vNOTES (*n* = 20).

	**Score**	***n* (%)**
How worried are you about scarring from “vNOTES” surgery? (Surveyed before surgery)	0 (No worry)	2 (10.0)
	1	5 (25.0)
	3	3 (15.0)
	4	1 (5.0)
	5	4 (20.0)
	7	2 (10.0)
	8	1 (5.0)
	10 (Very worried)	2 (10.0)
What was your overall surgical pain score? (Gas pain, surveyed after 1 week)	0 (No pain)	8 (40.0)
	1	3 (15.0)
	3	2 (10.0)
	5	1 (5.0)
	6	3 (15.0)
	7	2 (10.0)
	8	1 (5.0)
	10 (Severe pain)	0 (0.0)
What was your overall surgical pain score? (Low abdominal pain, surveyed after 1 week)	0 (No pain)	6 (30.0)
	1	3 (15.0)
	2	5 (25.0)
	3	2 (10.0)
	4	2 (10.0)
	8	2 (10.0)
	10	0 (0.0)
What was your overall surgical pain score? (Vaginal pain, surveyed after 1 week)	0 (No pain)	9 (45.0)
	1	4 (20.0)
	2	3 (15.0)
	3	4 (20.0)
	10 (Severe pain)	0 (0.0)
If you have surgery again, will you choose vNOTES again? (Surveyed 3–6 months after surgery)	10 (Absolutely)	19 (95.0)
	9	1 (5.0)
	0 (Never)	0 (0.0)
Would you recommend vNOTES to a friend? (Surveyed 3–6 months after surgery)	10 (Actively)	19 (95.0)
	9	1 (5.0)
	0 (Never)	0 (0.0)
What is your overall satisfaction after vNOTES? (Surveyed 3–6 months after surgery)	10 (Very satisfied)	19 (95.0)
	9	1 (5.0)
	0 (Disappointed)	0 (0.0)
How do you expect sexual life satisfaction will be after surgery? (Surveyed before surgery)	+10 (Improved)	0 (0.0)
	0 (No change)	7 (35.0)
	−3	2 (10.0)
	−6	2 (10.0)
	−7	1 (5.0)
	−8	2 (10.0)
	−10 (Decreased)	0 (0.0)
	N/A^a^	6 (30.0)
How has your sex life satisfaction changed after vNOTES? (Surveyed 3–6 months after surgery)	+10 (Improved)	0 (0.0)
	+3	1 (5.0)
	+2	1 (5.0)
	0 (No change)	3 (15.0)
	−2	1 (5.0)
	−3	3 (15.0)
	−4	1 (5.0)
	−9	1 (5.0)
	−10 (Decreased)	0 (0.0)
	N/A^a^	6 (30.0)
	F/U^b^ loss	3 (15.0)
How much are you willing to pay for vNOTES compared with conventional laparoscopic surgery? (Surveyed 3–6 months after surgery. The response unrelated to the surgery.)	No more	5 (25.0)
	< $499	5 (25.0)
	$500–999	5 (25.0)
	$1,000–1,499	2 (10.0)
	$1,500–1,999	0 (0.0)
	More than $ 2,000	0 (0.0)
	F/U^b^ loss	3 (15.0)

a *N/A, not applicable (no sexual activity for more than 3 months)*;

b *F/U, follow-up*.

## Discussion

The vNOTES method has been carried out at few institutions and considered as feasible and safe in gynecologic surgery compared with conventional laparoscopic surgery (3, 7–12). This study is the first report for vNOTES hysterectomy with patient survey results from South Korea. The study was conducted at Samsung Medical Center (SMC), which is a tertiary center and teaching University hospital. We have actively performed LESS or single-port access (SPA) laparoscopic surgery, which also utilizes embryonic opening of the umbilicus. This study is also useful for examining the learning curve of gynecologic surgeons using vNOTES because it analyzes surgical data from one gynecologist (T-J Kim).

We prospectively collected surgical data on 34 patients and survey data from 20 patients. The surgical data of 34 patients and survey data of 20 patients are small number. This is main limitation of this study. However, we collect the initial data of vNOTES prospectively and report co-relation pain score and port-installation time, learning curve, and complications related to vNOTES. We are currently studying on a large randomized controlled trial (RCT) on vNOTES.

There were three cases of conversion of surgical method. Two patients converted to classic VTH and one patient converted to LESS; the first case of three conversion was the third of 34 cases of vNOTES. The patient had no previous surgery or disease. We succeeded in installing the port at the vagina, but the operation method was changed to VTH because the camera field of view could not be secured due to bleeding around the cervix. The second case of three conversion was 17th of 34 cases and the patient had a previous Cesarean delivery. We tried to install the port at the vagina for 30 min but failed due to peritoneal adhesion. We barely completed VTH and then finished adnexal surgery by vNOTES after port-installation on vagina stump. The last case of conversion was to LESS.

There were two bladder injuries in this study and both patients had previous Cesarean delivery in another hospital several years before the current study. The first case was the 9th of 34 cases and it is the last case of conversion of surgical method. Bladder injury occurred while developing anterior vesicovaginal space for vaginal colpotomy due to anterior pelvic adhesion, and we changed the surgical method to LESS using the umbilicus. After hysterectomy, the bladder was repaired by suturing during LESS. The other case was the 26th of 34 cases. Similar to the first case, during the development of anterior vesicovaginal space, there was bladder injury. In this case, the injury was small and the operation was completed by vNOTES. Bladder repair was then performed through LESS. The urinary Foley catheter was maintained for 1 week after the surgery and removed after confirming healed bladder by cystography. Both patients showed restored bladder function. There were two cases of bladder injuries in six patients with previous Cesarean section.

While total laparoscopic hysterectomy (TLH) is associated with an increased risk of urinary tract injuries compared with other techniques, the overall risk of urinary tract injury of TLH is still relatively low, so previous Cesarean delivery should not be considered as a contraindication to either a VTH or TLH (13). We included the patient who had previous abdominal surgery including Cesarean delivery in this study. Bladder injury rate (5.9%, 2/34) in this study was higher than urinary tract injury (2.0%) in patients with hysterectomy by LESS at SMC (14). In laparoscopic surgery, the degree of adhesion can be seen before dissection. In vNOTES, however, vesicovaginal dissection was done without knowing the severity of adhesion, which is a major disadvantage of this approach. Therefore, surgeons should be more careful in vNOTES especially if patients had a previous surgical history such as Cesarean delivery.

There was one case of postoperative bleeding among the 34 patients. The patient was 48 years old, G1P1 and had no underlying disease. The patient underwent vNOTES for the treatment of leiomyoma with heavy menstrual bleeding and was discharged on postoperative day 2 uneventfully. Preoperative hemoglobin (Hb) was 13.0 g/dL and the postoperative Hb was 12.1 g/dL. On postoperative day 13, the patient visited the emergency department for abdominal pain. Hb decreased rapidly from 12.8 to 10.0 g/dL within 3 h after the emergency department visit. Computed tomography (CT) showed massive intraabdominal hemorrhage and suspected active bleeding. Therefore, we performed emergent diagnostic single-port laparoscopic surgery. A large amount of blood was detected in the abdominal cavity and bleeding was seen from the right paravaginal vessels. In vNOTES, we cannot check the surgical field after vaginal cuff closure. This is the major disadvantage of vNOTES. Therefore, it is recommended to carefully examine the operation site during irrigation and bleeding control before vaginal cuff closure.

Unlike conventional laparoscopy and transumbilical LESS, it is likely that vNOTES has less postoperative pain because there is no abdominal incision. Because pain is subjective, accurately assessing it with objectivity is not easy. Comparison with the SPA-TLH procedure in SMC (15) revealed that VAS at 12 h after operation was 2.84 in vNOTES and 3.6 in SPA-TLH. At 24 h after operation, VAS was 2.36 in vNOTES and 3.0 in SPA-TLH. VAS in vNOTES seemed to be lower than that in SPA-TLH in our hospital (15). Another reason may explain the reduced pain caused by vNOTES. We used advanced energy devices in all patients during vNOTES. Therefore, we expect the pain is reduced using the advanced energy device than the traditional tying-off method (16). In addition, instead of a vaginal retractor, a WR is used during the surgery, which allows for gentle retraction compared with the vaginal retractor. This may have reduced pain after surgery. However, further research on postoperative pain is needed.

In multivariate analysis, postoperative pain did not correlate with total operation time, but it showed a significant correlation with port-installation time. This suggests that post-operative pain may increase if longer time is spent to install the port due to the prolonged manipulations and interventions during the operation. The port-installation time likely decreases as the surgeon's skill reaches proficient levels, and the pain would decrease. We believe that vNOTES is easier to learn than single-port access laparoscopic surgery (17, 18), but further research is needed.

Unlike conventional laparoscopy or LESS, vNOTES does not require time for abdominal incision and closure. Instead, vNOTES needs port-installation time. Therefore, if the surgeon's skill to install the port reaches proficient levels, the overall operation time will be shorter than laparoscopy. Baekelandt et al. showed this in the Halon study (12). As the operation time shortens, the complications of anesthesia are reduced and the surgeon's fatigue is also decreased.

All surgical techniques require a learning curve for surgeon proficiency. All 34 cases in this study were performed by one experienced gynecologic surgeon. The port-installation time and total operation time reflected close to proficiency by the 10th case. Since this study were performed by an experienced gynecologic surgeon, the proficiency level by 10th case cannot be a standard. Depending on the individual surgical skill, the proficiency level may be reached in more than 10 cases. After reaching proficiency, we believe that postoperative pain, operation time and complications would be reduced as well.

The survey was conducted in the last 20 patients (Table 4). In the preoperative survey, many patients said that they were worried about scars from vNOTES surgery (although vNOTES does not cause visible scars). This may be because the patients did not have a good understanding of the operation of vNOTES, even if the medical staff might have provided an appropriate description of the operation to the patient, or patients were more sensitive about wounds than the surgeon thought. The response of survey unrelated to the surgery especially about financial question that was asked only after surgery.

The present study examined 34 patients only. This number of patients is insufficient to make a statistically meaningful conclusion regarding the safety of vNOTES. Furthermore, the surgeon who performed these surgical procedures is a skilled expert in minimally-invasive surgery with ample experience. Therefore, further studies are warranted to examine the safety of the surgical procedures. Adequately assessing and safely introducing new techniques in surgery is challenging and occurs slowly over many years. This relatively new surgical procedure is still at its exploration stage. Experience with the procedure is still scarce, and outcomes with larger numbers of patients are needed. Data should be captured systematically for every patient having the procedure, especially to ensure that adverse outcomes are documented. In this aspect, the present study is invaluable especially in that it reported the initial learning curve of the procedures along with surgical complications that occurred.

## Conclusion

We analyzed initial experiences with vNOTES at a single center in South Korea. Although there were three complications and two intraoperative conversions to VTH, we believe, vNOTES offers advantages to patients and surgeons. More surgical techniques will be developed in vNOTES.

## Data Availability Statement

The raw data supporting the conclusions of this article will be made available by the authors, without undue reservation.

## Ethics Statement

The studies involving human participants were reviewed and approved by Institutional Review Board (IRB) of Samsung Medical Center (SMC 2018-06-011-001). The patients/participants provided their written informed consent to participate in this study.

## Author Contributions

T-JK: conception and design. JN: administrative support. M-SK: provision of study materials or patients, collection and assembly of data, data analysis, and interpretation. M-SK and JN: manuscript writing. All authors: final approval of manuscript.

## Conflict of Interest

The authors declare that the research was conducted in the absence of any commercial or financial relationships that could be construed as a potential conflict of interest.
