# Supplementary material for: Hysterectomy and Adnexal Procedures by Vaginal Natural Orifice Transluminal Endoscopic Surgery (VNH): Initial Findings From a Korean Surgeon
Source: Front Med (Lausanne). 2021 Feb 22;7:583147. doi: 10.3389/fmed.2020.583147 (PMC7937714; doi:10.3389/fmed.2020.583147)
Supplement: Supplementary file 1 [file Table_1.DOCX]

**Supplement**

**Table S1.** Correlation between (A) port-installation time and continuous variables and (B) total operation time and continuous variables

(A)

| Variable | Correlation | *P* value |
| --- | --- | --- |
| Age | 0.3090 | 0.1513 |
| Body weight | 0.0749 | 0.7342 |
| Height | -0.0040 | 0.9857 |
| BMI | 0.0479 | 0.8282 |
| Number of vaginal delivery | -0.0887 | 0.6875 |
| Uterine weight | 0.2075 | 0.3540 |
| Postoperative Hb changes | 0.1464 | 0.5052 |

(B)

| Variable | Correlation | *P* value |
| --- | --- | --- |
| Age | 0.2489 | 0.2202 |
| Body weight | 0.2661 | 0.1888 |
| Height | 0.1290 | 0.5301 |
| BMI | 0.1018 | 0.6207 |
| Number of vaginal delivery | 0.0879 | 0.6694 |
| Uterine weight | 0.3475 | 0.0961 |
| Postoperative Hb changes | 0.2004 | 0.3264 |

**Table S2.** Correlation between (A) port-installation time and variables and (B) total operation time and variables.

(A)

|  |  | N | Mean | Median (range) | *P* value |
| --- | --- | --- | --- | --- | --- |
| VAS, postop | VAS < 4 | 15 | 12.9 | 12 (4–27) | 0.0308 |
|  | VAS ≥ 4 | 7 | 21.0 | 18 (12–32) |  |
| VAS, 6 h | VAS < 4 | 16 | 15.7 | 15 (4–35) | 0.8532 |
|  | VAS ≥ 4 | 6 | 17.7 | 12.5 (12–30) |  |
| VAS, 12 h | VAS < 4 | 18 | 16.9 | 15.5 (4–35) | 0.1138 |
|  | VAS ≥ 4 | 4 | 10.3 | 12 (4–13) |  |
| VAS, 24 h | VAS < 4 | 21 | 16.3 | 15 (4–35) | 0.9128 |
|  | VAS ≥ 4 | 2 | 16.5 | 16.5 (12–21) |  |
| CO_2_ pressure (mmHg) | < 12 | 17 | 13.9 | 13 (4–30) | 0.0731 |
|  | ≥ 12 | 6 | 23.0 | 26 (4–35) |  |
| History of vaginal delivery | No | 7 | 15.7 | 13 (9–30) | 0.8933 |
|  | Yes | 16 | 16.6 | 15 (4–35) |  |

(B)

|  |  | N | mean | median (range) | *P* value |
| --- | --- | --- | --- | --- | --- |
| VAS, postop | VAS < 4 | 17 | 83.8 | 82 (43–132) | 0.5798 |
|  | VAS ≥ 4 | 8 | 88.0 | 89.5 (57–125) |  |
| VAS, 6 h | VAS < 4 | 19 | 91.2 | 89 (43–132) | 0.0800 |
|  | VAS ≥ 4 | 6 | 68.8 | 63.5 (52–92) |  |
| VAS, 12 h | VAS < 4 | 21 | 90.1 | 92 (43–132) | 0.1195 |
|  | VAS ≥ 4 | 4 | 67.3 | 63.5 (57–85) |  |
| VAS, 24 h | VAS < 4 | 24 | 87.5 | 87 (43–132) | 0.4703 |
|  | VAS ≥ 4 | 2 | 73.0 | 73 (64–82) |  |
| CO_2_ pressure (mmHg) | < 12 | 19 | 81.1 | 76 (43–132) | 0.0993 |
|  | ≥ 12 | 7 | 101.0 | 105 (57–121) |  |
| History of vaginal delivery | No | 8 | 84.4 | 79 (52–125) | 0.8895 |
|  | Yes | 18 | 87.3 | 87 (43–132) |  |
